# M Cells: Intelligent Engineering of Mucosal Immune Surveillance

**DOI:** 10.3389/fimmu.2019.01499

**Published:** 2019-07-02

**Authors:** Andrea Dillon, David D. Lo

**Affiliations:** Division of Biomedical Sciences, School of Medicine, University of California, Riverside, Riverside, CA, United States

**Keywords:** mucosal immunity, epithelium, innate immunity, endocytosis, Inflammatory Bowel Disease

## Abstract

M cells are specialized intestinal epithelial cells that provide the main machinery for sampling luminal microbes for mucosal immune surveillance. M cells are usually found in the epithelium overlying organized mucosal lymphoid tissues, but studies have identified multiple distinct lineages of M cells that are produced under different conditions, including intestinal inflammation. Among these lineages there is a common morphology that helps explain the efficiency of M cells in capturing luminal bacteria and viruses; in addition, M cells recruit novel cellular mechanisms to transport the particles across the mucosal barrier into the lamina propria, a process known as transcytosis. These specializations used by M cells point to a novel engineering of cellular machinery to selectively capture and transport microbial particles of interest. Because of the ability of M cells to effectively violate the mucosal barrier, the circumstances of M cell induction have important consequences. Normal immune surveillance insures that transcytosed bacteria are captured by underlying myeloid/dendritic cells; in contrast, inflammation can induce development of new M cells not accompanied by organized lymphoid tissues, resulting in bacterial transcytosis with the potential to amplify inflammatory disease. In this review, we will discuss our own perspectives on the life history of M cells and also raise a few questions regarding unique aspects of their biology among epithelia.

## Introduction: An Unusual Cell With a Unique Job

In the intestine, as well as in related mucosal tissues such as the upper airway and lung, the immune system is dependent on mechanisms that can both retain the effective barrier function of the mucosal epithelium, while enabling an efficient surveillance of luminal microbes. There have been a variety of mechanisms described whereby luminal components can be captured for the purposes of immune surveillance. These include the extension of dendritic cell processes in between enterocytes, enabling the capture of bacteria for direct uptake by dendritic cells (1, 2), and the ability of goblet cells to provide a conduit for small molecular weight soluble proteins to pass across the epithelial barrier (3). However, one of the most interesting mechanisms described for luminal surveillance is the capture and transcytosis of microparticles by specialized epithelial M cells (4–7).

**Figure 1:**
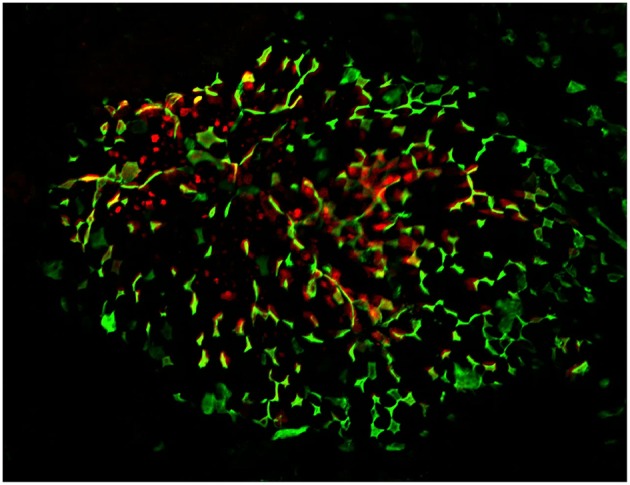
Confocal microscopy “en face” image of Peyer's patch, stained for M cells using UEA-1 lectin (green), with cytoplasmic expression of PGRP-S-dsRed reporter transgene evident below the surface of the follicle epithelium. Radial spoke distribution of M cells indicates origins in lineage-committed crypt stem cells.

**Figure 2:**
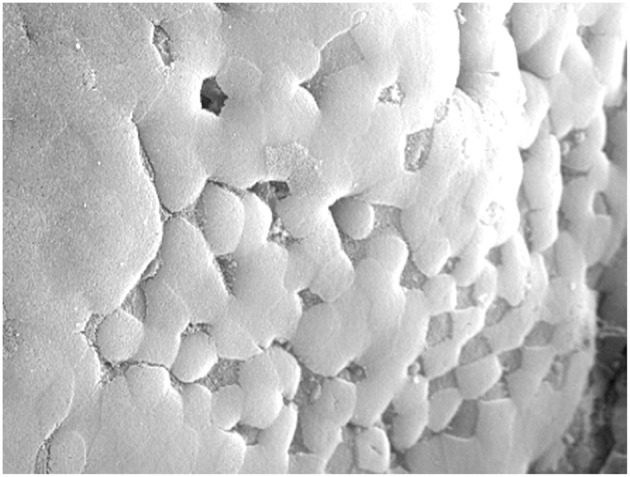
Scanning Electron Microscopy image of Peyer's patch showing the uniform apical brush border surface of follicle epithelium, interspersed with M cells lacking microvilli which appear as “divots” in the surface.

M cells are mainly found in the epithelium overlying organized mucosal lymphoid tissues (Figures 1, 2); despite the fact that they appear under a variety of circumstances, they still display a set of common morphologic and functional features that helps us classify them as M cells. In this discussion, we will describe the known features of the best-known M cells, identified among the epithelium of Peyer's patches (PP), Isolated Lymphoid Follicles (ILF), Colonic Patches (CP), and Nasopharyngeal Associated Lymphoid Tissues (NALT). We will also discuss Villous M cells, a related M cell-like phenotype, that differ from the other M cells in anatomic location and function. While we attempt to be broadly inclusive in this discussion, we apologize for not being fully comprehensive.

We should be clear from the outset that M cells are not antigen presenting cells in the conventional “dendritic cell” sense, but rather they are “antigen delivery cells” that transfer luminal particles and antigens to bona fide dendritic cells for antigen presentation. In that role, they may act as neutral brokers without direct influence on the character of the immune response, leaving that function to the dendritic cells and other immune effector cells and cytokines. Thus, M cell targeted antigen delivery can induce either mucosal immune responses (8–12) or mucosal tolerance (13, 14), depending on the response of underlying mucosal immune cells. The orientation of the polarized M cell toward the lumen is designed for capturing immunogenic particles at its apical surface, acquiring only from the mucosal lumen; this is in marked contrast to solid tissues, where lymphatics guide antigens and activated phagocytic cells from across the tissue bed into the draining lymph nodes. In this way M cells can be viewed as a functional equivalent of lymphatics for mucosal lymphoid tissues; in the absence of M cells, mucosal immunity would be handicapped without an efficient mechanism for antigen monitoring. Indeed, one study with a model of M cell deficiency confirmed impaired mucosal immune responses (15).

The transcytosis function of M cells requires a whole suite of novel cellular specializations, including capture of luminal particles, transport of cargo across the mucosal barrier, and delivery to the submucosal tissues. The surveillance requirements in the mucosal lumen are specifically directed at microparticles such as viruses and bacteria, since they comprise the main threats to the host requiring an appropriate immune response. Other mechanisms are available for uptake of soluble antigens such as food antigens (or perhaps microbial toxins), but their impact on immune surveillance may be rather different.

The anatomy of M cells is also optimal for capturing invasive microbes where they pose the greatest threat. For example, in the mouse, Peyer's patches are found scattered along the length of the small intestine, with additional lymphoid tissue in the cecum, and they are complemented by a scattering of colonic patches (16). In the upper airway, NALT is found along the floor of the nasal passage, serving a similar sentinel purpose as human tonsils. In the lung, lymphoid aggregates can be found at bronchial branch points (17).

Unfortunately, at this point we are handicapped by the differences in mouse vs. human anatomy, and this discussion will be largely limited to mouse M cells, since more information is available from experimental studies on the mouse mucosal immune system. As examples of the challenges presented by these differences, we point out that in human tonsils, the lymphoid tissues are covered by stratified squamous epithelium instead of the simple ciliated epithelium found over NALT; moreover, despite their general histological similarities to PP M cells, tonsil M cells are present within crypt invaginations (18, 19), so less is known about how these cells function. Similarly, in the lungs, less is known about lymphoid tissues and any potentially associated M cells in mice, though in one study, histological evidence for M cells has been described at bronchial epithelium branch points overlying small lymphoid aggregates (17); however, with normal vivarium housing, mouse lungs do not generally develop bronchus-associated lymphoid tissues (BALT) (20–23).

## The Basic M Cell and Interacting Neighbors

The prototypic Peyer's patch M cell is an epithelial cell, derived from stem cells in the intestinal crypt, and has a lifespan similar to neighboring enterocytes [~5 days, Creamer (24)]. The mature M cell has several morphological features that distinguish it from other epithelial cells. First, M cells lack apical microvilli resulting in a “microfold” or “membranous” appearance, also providing the “M” of the M cell name (25–27). Second, the M cell is not a lone actor, and generally has a basolateral pocket that usually contains a B lymphocyte, though T cells and myeloid cells may also be present.

**Figure 3:**
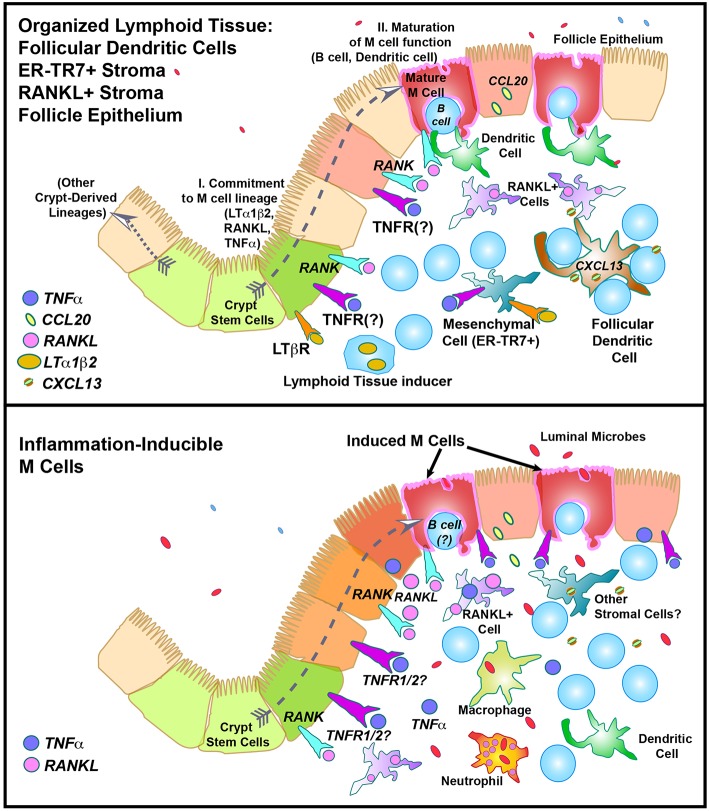
Speculative model of M cell development in “Constitutive” **(Top)** or “Inflammation-inducible” **(Bottom)** settings. In the top figure, the “Two Step” model of M cell development is illustrated, showing “I. Lineage commitment,” and “II. Maturation of function”.

The basolateral pocket B cell is actually quite an important partner in M cell biology, though it is not entirely clear why. Studies have shown that the B cell, normally a relatively short-lived cell, appears to remain associated with the M cell for the life of the M cell (28, 29). Interestingly, although B cells are responsible for IgA production, a hallmark of mucosal immunity, the basolateral pocket B cell does not appear to be destined to become an IgA-producing cell, so its importance to M cell development is curious. This B cell interacts directly with the M cell and is required for the maturation of M cell function, at least in the Peyer's patch. This interaction is at least partly dependent on M cell expression of CD137, as CD137 knockout mice fail to form B cell basolateral pockets, and the M cells do not develop transcytosis function (30). This is consistent with a two-step model of M cell differentiation (Figure 3, top; see also below), with initial commitment to the M cell lineage followed by a CD137-CD137L interaction of M cells with CD137-activated B lymphocytes or dendritic cells for functional maturation.

These earlier studies suggest that B cells are an absolute requirement in the development of Peyer's patch M cell function but not lineage commitment, but are B cells always required for M cell development and function? Some studies show that under specific conditions, M cell phenotypes can develop in the absence of B cells. For example, in organoid cultures (31–33), B cells and myeloid cells are not present, yet functional M cells can be induced by the addition of the cytokine RANKL. In mice lacking B cells, M cells were dramatically reduced although not entirely absent (28). These results and others suggest that there may several functional subsets of M cells (see below), or intermediate forms that can develop after lineage commitment, albeit with variable functional ability.

**Figure 4:**
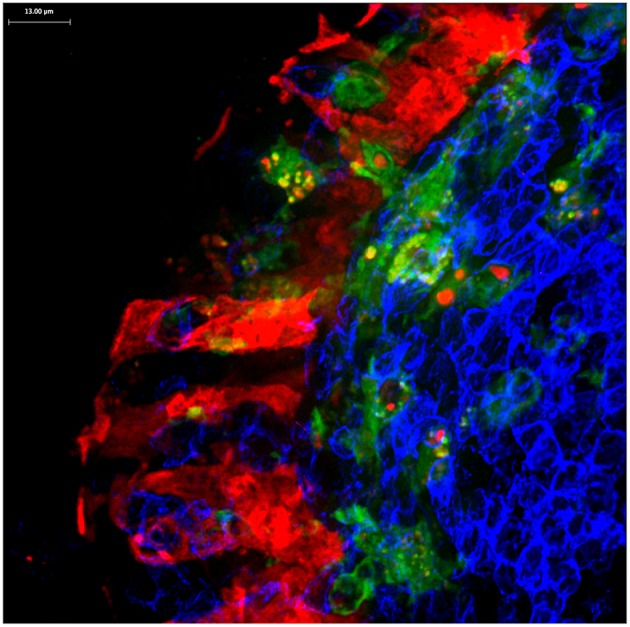
Confocal microscopy image of Peyer's patch showing PGRP-S-dsRed+ M cells (red) intimately associated with CX3CR1-EGFP+ subepithelial dome dendritic cells (green). M cell-derived dsRed+ cytoplasmic vesicles are evident in the dendritic cells.

In Peyer's patches, M cells are also closely associated with dendritic cells (34–36), which rapidly take up the antigenic cargo released by M cells (Figure 4). Whether this association is mediated by any adhesion molecules or other interactions is not clear, and such interactions appear to not be required for M cell development, as clodronate treatment to reduce myeloid cells had no apparent effect on M cell development (36). The close association with dendritic cells likely insures an efficient transfer of cargo, but the fate of the transcytosed cargo may actually be determined by the receiving dendritic cells. For example, while both bacteria and latex microparticles can both be efficiently transcytosed by Peyer's patch M cells, bacteria are preferentially taken up by the underlying dendritic cells, while latex particles appear to be left in the extracellular space of the subepithelial dome region (36). In other words, M cells can transcytose nearly any microparticle, but the impact on immune recognition is still a team effort.

M cells also appear to produce a different type of cargo for uptake by dendritic cells. In mice expressing an M cell-specific reporter transgene (PGRP-S-dsRed), M cell cytoplasm is loaded with the fluorescent protein dsRed. Interestingly, microvesicles containing this cytoplasmic dsRed were found to be produced at the basolateral side of the M cells, and were readily taken up by dendritic cells (36) (Figure 4); when clodronate treatment reduces the available dendritic cells, the M cells developed cytoplasmic projections that appear to be the source of these dsRed+ microvesicles. Since these vesicles did not contain transcytosed bacterial particles, these may represent a parallel mechanism for M cells to deliver cytoplasmic antigens to dendritic cells. These antigens may include antigens or fragments from obligate intracellular microbes, or components from viruses that may have uncoated in the M cells.

As noted, M cells are associated with organized mucosal lymphoid tissues, and these tissues have a characteristic organization below the epithelium. In the same manner that M cells are polarized and oriented toward the mucosal lumen, the lymphoid tissue is also similarly oriented toward the M cells and associated epithelium. From the perspective of the M cells, this relates to the M cell as the main source of antigenic stimulus, but it is also relevant to the importance of inducing adjacent crypt stem cells that give rise to the lineage-committed M cell precursors. Electron microscopy studies on Peyer's patches found that the crypts giving rise to FAE cells showed morphological differences from crypt cells producing conventional enterocytes (37–39), suggesting that the crypt stem cells are in some way already committed to produce cells, suggesting the existence of a distinct M cell lineage. This commitment is associated with expression of a set of M cell-specific genes, including gp2 (40–42), Spi-B (31, 43), PGRP-S (44, 45), TRAF6 (32), and CD137 (30), and at this stage the committed cells are also paired for life with a B lymphocyte in a basolateral pocket. Among these genes, Spi-B appears to be a transcription factor regulating the M cell developmental program.

As with enterocytes emerging from the intestinal crypt on their way to the tips of the villi, M cells also migrate from the crypt across the face of the PP FAE to the center of the FAE. Because of the distribution of crypts at the margins of the FAE, the M cell migration results in a characteristic radial spoke-wheel arrangement (Figure 1). This pattern seems to be under the influence of Jagged1—Notch interactions. M cells appear to express Jagged1, so that lateral inhibition effects insure that the M cells are not clustered as they move across the FAE, resulting in a superimposed checkerboard pattern (46). This effect results in a uniform distribution of M cells across the FAE that may be in some way important to the efficient capture of luminal microparticles that may drift across the FAE.

## M Cell Microparticle Capture: The Hunt for Capture Receptors

Among epithelial tissues, it is uncommon for cells to capture and transport molecules or microparticles from the apical face for transport and release at the basolateral side; thus, M cells present a rather unique biology among epithelial phenotypes. It is more common for epithelial cells to secrete material into the lumen, such as secretory granules (e.g., zymogen). In the case of transcytosis of molecules (i.e., combining uptake and delivery), mucosal epithelium is able to deliver IgA from the lamina propria into the mucosal lumen using the poly Ig receptor (PIgR) (47, 48), but this is a specialized process involving cleavage of the poly Ig receptor and secretion of the dimeric IgA in a complex with secretory component.

Are there candidate mechanisms available for M cell microparticle uptake and delivery at the basolateral side? For apical to basolateral transport in the intestine, there is one notable example: the neonatal Fc Receptor (47, 48), which takes up luminal immunoglobulin for uptake of immunoglobulin from maternal milk, but this applies to a very specific molecular cargo. Given the need for the epithelium in the intestine to maintain an intact barrier to microbial invasion, there are few other obvious candidates for M cell transcytosis mechanisms. Thus, for M cell particle capture and transcytosis, studies have sought to identify M cell-specific capture receptors that might explain this process.

The search for M cell capture receptors implies a specificity for pathogen particles that might be similar to Toll-like Receptors, able to bind a subset of pathogen-associated molecules. Yet in principle, any pathogen-specific capture receptors would be unable to distinguish between commensal vs. pathogenic microbes, in the same way the mucosal immune response in general cannot make this distinction. We are then left with the question of whether M cells need to identify the physical or chemical properties of microparticles likely to be most relevant to pathogenesis, or at least important to trigger the initiation of mucosal immunity.

The considerations here are theoretical, but meanwhile a number of studies have suggested that actual specific capture receptors do exist and they help define some of the functional capabilities of M cells. As we will discuss, there will be some additional work needed to help sort out whether all of these mechanisms are compatible in the same cell.

The search for capture receptors were based on differential M cell specific expression of genes that may encode transmembrane molecules (44, 49). From this approach, a few candidate receptors have been described. Among the first was the tight junction protein Claudin-4, which was notable for increased expression in mouse Peyer's patch M cells, and histological evidence that it was redistributed from tight junctions to the cytoplasm of both mouse and human M cells (49). Tight junctions and M cell transcytosis will be discussed in more detail later on.

More comprehensive surveys of M cell-specific gene expression (50–52) yielded a number of other candidate capture receptor genes. One notable example is the protein glycoprotein 2 (gp2) (40, 41). Studies suggested that gp2 specifically bound FimH, a fimbria protein on *Salmonella*. A knockout of gp2 appeared to abrogate M cell function, or at least uptake of *Salmonella*.

Earlier studies have also identified an IgA-binding activity in mouse Peyer's patch M cells, though the gene or protein providing this activity has not been identified (53). An IgA binding activity might be a useful surveillance mechanism, since secretory IgA should be capable of binding both pathogenic and commensal intestinal microbes (54), and M cell uptake through this mechanism might help monitor any changes in the resident intestinal microbiome.

The specificity of M cell microparticle uptake is not entirely up to the M cell, as some microbes provide their own targeting ligands for M cell uptake. For example, *Yersinia enterocolitica* expresses an adhesion molecule, Invasin, that binds to beta-1 integrin that is curiously redistributed to the apical surface of M cells (55–57), in contrast to its normal location on the basolateral side of conventional enterocytes. Uptake of *Yersinia* by this mechanism appears to be through macropinocytosis (58), which may be a unique pathway through M cells. Other microbes appear to target M cells for invasion (59, 60), though specific mechanisms have not yet been identified. As we will discuss later, viruses also have their own approaches to take advantage of available molecules on M cells.

Other bacteria with specific pathogenicity mechanisms also appear to provide their own machinery. *Salmonella*, already mentioned above, have adhesion and invasion proteins encoded in a Type Three Secretion System (TTSS) SPI-1 locus that, in addition to providing capabilities for invading enterocytes, may specifically provide enhanced capabilities mediating uptake by Peyer's patch M cells (61–63); however, another study suggested that SPI-1 mutants remain fully capable of preferential M cell uptake (64), and as discussed in the next section, non-specific uptake mechanisms also provide some ambiguity to this question.

Studies on *Salmonella* invasion have also suggested an unusual phenomenon in which the TTSS mechanism may also induce enterocytes to develop acutely into M cells (61). In another infection model, inoculation of rabbits with *Streptococcus* appears to rapidly induce new M cell development (37) at the perimeter of the FAE, and increased M cell transcytosis activity was also observed (65). We mention these phenomena here because they appear to be directly induced by infection rather than by indirect mechanisms or chronic inflammation as will be discussed below.

## Morphology Is Function: Microvilli and Electrostatics

Despite the previous discussion on M cell specific capture receptors, it is important to point out that most studies on M cell transcytosis have relied on the observation that latex microparticles are readily taken up by Peyer's patch M cells [e.g., Gebert et al. (65)]. Since latex does not present any pathogen-like activity, this phenomenon suggests that M cells have a kind of “particle-agnostic” activity. This also emphasizes the notion that M cells may be particularly specialized for microparticle uptake, whereas soluble proteins or other small molecules do not appear to be taken up as readily by M cells unless specifically targeted. Thus, most food components after digestion are likely to be ignored by M cells while for reasons discussed below, microbes and certain microparticles may have common physico-chemical properties attractive to M cells. Does this activity suggest unique M cell specializations?

A key characteristic of M cell morphology is the absence of apical microvilli in the intestine and absence of cilia in the upper airway (Figure 2). This feature could conceivably be helpful for luminal microparticles to bind apical capture receptors, but recent studies provided evidence for a far more useful consequence for M cell transcytosis.

Epithelial microvilli are complex structures with a central actin/myosin filament (66, 67), with an array of glycoproteins on the apical membrane; the tips of the microvilli are also cross-linked by protocadherins (67), helping to insure a regular orderly array of microvilli. To assess the consequences of blocking microvillus formation, Caco-2BBe cells, a subclone of Caco-2 cells selected for their ability to form mature microvilli, were transfected with a mutant fusion protein to block microvillus formation (68).

The transfected cells not only lacked mature microvilli, they also showed a significant reduction in the amount of carbohydrate associated with glycoproteins (68). The surface charge of the epithelial cells is in large part dependent on the carbohydrate moieties presented by surface glycoproteins, resulting in a strong net negative charge, to reductions in surface glycoproteins would be expected to reduce the net surface negative charge. Would this change affect interactions with luminal microparticles?

To test this, the microvilli-minus cells were tested for their ability to capture a series of bacterial particles in laminar flow chambers. The bacterial particles were assayed for their surface charge as well. From these studies, a pattern emerged where the binding of bacteria under laminar flow conditions was greatly enhanced for the microvilli-minus cells, and the degree of binding was directly proportional to the surface charge of the particles (68). The scenario developed from these results proposes that negatively charged bacterial microparticles are unable to bind normal microvilli-positive cells due to electrostatic repulsion. In contrast, microvilli-minus cells with lower negative surface charge will not repel the bacteria, so that these particles can move close enough to the apical membrane to allow Van der Waals forces to capture the particles (or allow capture receptors or adhesion molecules to engage). In this way we can view M cell particle uptake as a kind of “dust bunny” collector, as particles are electrostatically repelled from conventional enterocytes with mature microvilli, bouncing into M cell traps.

It turns out that latex microparticles are also strongly negatively charged, in the same range as Gram-positive bacteria. Thus, their uptake may rely on a similar mechanism, where electrostatic repulsion explains their relative preference for M cells. This may provide at least a partial explanation for the promiscuity of M cell uptake and transcytosis, but clearly a few more details are needed to explain the binding and endocytosis mechanisms. For example, it would be helpful to know whether Van der Waals interactions are alone sufficient to trigger particle endocytosis once microparticles settle at the apical membrane.

The apparent promiscuity of M cell uptake also raises questions about the notion that specific capture receptors are important to M cell function. While the reduction in electrostatic repulsion can allow microparticles to get close enough to the M cell apical membrane to interact with surface capture receptors, are capture receptors even necessary at this point? That is, in the absence of the capture receptor, microparticles might still be likely to be endocytosed regardless of any interaction with a specific receptor. In that context, how do we view the studies on mutant mice lacking specific capture receptors; would electrostatic mechanisms alone be sufficient to compensate for the loss of the specific receptor?

## M Cells, Endocytosis, and Tight Junction Proteins

Once an M cell captures a microparticle, it faces the task similar to the worker ant that has to carry a huge cargo. In this case, the M cell has to endocytose a particle of unusual size. Receptor-mediated endocytosis mechanisms (e.g., clathrin- or caveolin-mediated uptake) characteristically spontaneously assemble into endosomes in the 50–150 nm range (69), but bacteria can be upwards of 1 to 10 microns across, far larger than can be handled by conventional endocytic mechanisms. Does this function require additional specializations?

The earliest studies on M cell-associated gene regulation provided some of the first clues; in a gene expression profiling study of Peyer's patch epithelium, the tight junction protein Claudin-4 was found to be upregulated in both mouse and human M cells (49). More intriguing, the protein was redistributed from the tight junctions to the cytoplasm in immunostained sections, suggesting that Claudin-4 was re-assigned a new role in M cells. If Claudin-4 had a new role in M cells, it might be to participate in particle endocytosis. To test this possibility, phage display selection of potential short peptide ligands was used to identify a peptide motif that turned out to closely resemble the sequence of the binding domain of the *Clostridium perfringens* enterotoxin (CPE), also known to bind to an extracellular domain of Claudin-4 (70, 71). When this “targeting” peptide was attached to synthetic microparticles, it proved to mediate effective and rapid M cell uptake. Moreover, when attached to an engineered recombinant influenza hemagglutinin protein antigen, it provided a significant boost to mucosal IgA antibody responses when it was administered as a mucosal vaccine (10). Together, this provided an argument that tight junction proteins such as Claudin-4 are recruited to endosomes as part of the capture of luminal microparticles.

How can tight junctions participate in M cell surveillance? One possibility is that incorporation of the tight junction proteins into the endosome membrane, especially the transmembrane proteins, can alter the geometry of the endosome, allowing it to accommodate large cargo such as bacterial particles even when mediated by clathrin or caveolin (72–74). There is little information on any tight junction role in large particle endocytosis, but interestingly the small GTPase Rab13 was found to be upregulated in gene discovery studies on M cell-associated genes (49). Rab13 is thought to be responsible for guiding the transport of tight junction proteins from the trans-Golgi to the tight junctions (75–77); given the apparent redistribution of tight junction proteins such as Claudin-4, it is possible that Rab13 is also re-assigned in M cells to guide them to the early endosomes.

Other candidate proteins involved in endocytosis may also play a role in M cell particle uptake. For example, it has been suggested that caveolin is involved in tight junction protein recycling in response to the cytokine TNFα (78); thus, caveolin and/or tight junction proteins (and Rab13) might be pulled into M cell endosomes.

Interestingly, viruses are known to rely on several tight junction proteins as cellular receptors. Examples include JAM-A [receptor for *Reovirus*, (79, 80)], occludin [receptor for *Hepatitis C* virus, (81, 82)], and CAR [the *Coxsackie-Adenovirus* receptor (83, 84)]. In the case of *Reovirus*, the use of the JAM-A protein as a receptor is significant since M cells were already known to be the main entry point for this virus to infect mice. This raises the intriguing possibility that if these tight junction proteins are also recruited into M cell endosomes, viruses might take advantage of the tight junction proteins as cellular receptors, not only for infection of epithelial cells, but also to use these molecules in M cell endosomes to gain more efficient entry into the body.

If it turns out that a whole array of tight junction proteins are indeed re-assigned a rather different role in the service of M cell transcytosis, there remains a lot yet to be explained. For example, are tight junction proteins only involved in early endocytosis, and soon after recycled back to the tight junctions? Alternatively, do the tight junction proteins accompany the cargo all the way to the basolateral side of the M cell? Finally, assuming there is an exocytosis at the end of the journey, does that process recruit yet another novel cellular machinery?

## Organized Lymphoid Tissues and the Life History of “Constitutive” M Cells

Up to this point, we have been discussing M cells in isolation, but we need to consider them in context, since the fruit of their efforts (transcytosis) might have good or bad ends, depending on the setting. M cells are conventionally found in the epithelium overlying organized lymphoid tissues, and for good reason. The organization of mucosal lymphoid tissues is optimally designed to transfer the M cell cargo to a gauntlet of dendritic cells with dendrites that are intimately associated with the M cells (34, 36, 85). Below that layer are the B lymphocyte follicles, also awaiting the delivery of antigens.

This organization is important for a few reasons. Antigen is delivered to waiting dendritic cells with rapid uptake and processing for presentation to the T lymphocytes in the subepithelial zone. Any antigens able to drift past the dendritic cells find their way to the FDC and naïve B lymphocytes in the follicles. Together, these layers of cells establish an effective gauntlet that provides a level of protection (albeit imperfect) from microbial invasion.

The organization of this gauntlet is established by the differentiation of specialized stromal cells that not provide a scaffolding for the organized tissue, as well as cytokines and chemokines that induce migrating cells to settle into specific compartments. The FAE produces the chemokine CCL20 (86, 87) to attract subepithelial dendritic cells and B cells (88, 89), while Follicular Dendritic Cell produce CXCL13 to recruit B cells into follicles (90). Other stromal cells express CCL21 to recruit dendritic cells and T cells into the interfollicular zones (91). Yet another subset of stromal cells in a discrete layer below the FAE expresses the cytokine RANKL (92, 93). This cytokine has been shown to correlate with M cell induction, and in organoid cultures, addition of exogenous RANKL was sufficient to induce functional M cell development (31, 33, 94–96).

This pattern of immune and stromal cell organization is found in PP, ILF, CP, and to some degree in NALT (and presumably in iBALT). Since PP, CP, and NALT development is initiated in the perinatal period, these might be considered part of a canonical “constitutive” pattern of development of mucosal lymphoid tissues. As noted above, initiation of the lineage commitment is presumably dependent on LTi cells, one of the Innate Lymphoid Cell (ILC) subsets (97), expressing cytokines such as lymphotoxin α1β2 heterotrimers that bind to the lymphotoxin-beta receptor (LTβR) on target cells (in this case, FRC); blockade of the LTβR prevents development of organized lymphoid tissues (98), at least the constitutive lymphoid tissues.

**Figure 5:**
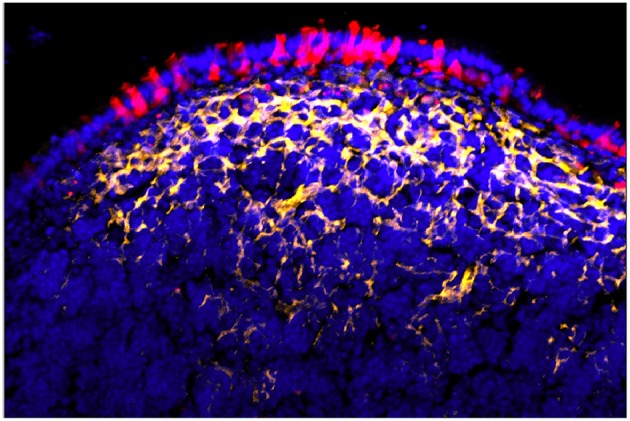
Peyer's patch showing FAE M cells (red) with underlying array of RANKL+ stromal cells (yellow). Note that RANKL staining is strongest under the main concentration of M cells rather than near adjacent crypt stem cells [reprinted from Parnell et al. (96) with permission].

The effect of organized lymphoid tissues, LTi, and local RANKL production on the adjacent crypt stem cells produces a variety of cell phenotypes in the Follicle Associated Epithelium (FAE) (38); one or more of the TNF related cytokines produced by LTi (as well as FRC and other cells) may account for FAE being uniformly positive for NF-κB activation and relB expression (99–101). Yet this global activation of FAE does not explain the sequence of events resulting in crypt cell commitment of a specific subset of enterocytes to M cell production. Moreover, while organoid cultures confirm the requirement for RANKL in M cell induction, the location of RANKL+ stromal cells in Peyer's patches is curious. Instead of being located immediately adjacent to the crypts, they appear to be predominantly in the subepithelial dome region (96) (Figure 5). Thus, while RANKL may have a role in crypt cell induction and lineage commitment (Step 1 in the two step model), the anatomy of RANKL expression suggests that RANKL, instead of being simply an early inducer of crypt stem cells, may also provide reinforcement of an early M cell lineage commitment (induced how?), or a persistent signal that results in a late lineage commitment.

How do these observations affect the two-step model of M cell development? (Figure 3) Considering for a moment the implications of late (rather than early) RANKL-induced M cell lineage commitment: late signals may help coordinate additional parallel events such as the pairing of nascent M cells with B cells forming the basolateral pocket (CD137-dependent), the role of Jagged-1/Notch signaling to coordinate the distribution of M cells across the FAE, and possible pairing with sub-epithelial dendritic cells. Another reason to make this distinction is that if the same events are not closely coordinated as in organized mucosal lymphoid tissues, would less coordinated RANKL production in the absence of organized lymphoid stromal tissue have different consequences in M cell induction?

## An Abbreviated Life History: Transdifferentiation of M Cells

Having described the specific consequences of M cells and their particle delivery within organized lymphoid tissues, we compare this with M cell phenotypes outside of this context. Cells with the M cell morphology can be induced in a few settings that place them outside the conventional “constitutive” pattern. For example, in the mouse, treatment with cholera toxin appears to induce rapid (24–48 h) development of new M cells in NALT epithelium, and in the intestinal villi (45).

The rapid induction of M cells in the NALT epithelium is distinct from the case in intestinal epithelium, as NALT epithelium is predominantly ciliated airway epithelium (unlike human tonsils), and instead of production from crypt stem cells, ciliated airway epithelium is generated from basal cells (92, 94, 95). The transcription factor Foxj1 determines the genetic program for ciliated airway epithelium, and studies in the bronchial epithelium provided evidence that these cells are terminally differentiated. Thus, chemical damage leading to the development of Clara cells was due to production from basal stem cells rather than from transdifferentiation of extant ciliated airway epithelium.

M cell induction in the NALT however, seemed to violate that rule. In mice carrying a Foxj1-EGFP reporter and the M cell specific PGRP-S-dsRed reporter, an unusual subset of EGFP+dsRed+ epithelial cells in NALT epithelium appeared to be a latent intermediate epithelial cell population (45, 102). When treatment of mice with cholera toxin induced an increase in NALT M cells, these appeared to be produced within 24–48 h by conversion of the EGFP+dsRed+ cells to EGFP-dsRed+ M cells. Thus, at least in this case, the rapid production of M cells appeared to be an example of transdifferentiation from Foxj1+ cells. We note that NALT may not be the same as inducible lymphoid tissue in lung bronchi (inducible Bronchus Associated Lymphoid Tissue, or iBALT (20–22), so while M cells have been described in aging mouse bronchial epithelium (17) it has not yet been reported whether M cells are associated with iBALT, or whether they develop in a distinct manner.

The induction of NALT M cells by cholera toxin has a parallel in the villous epithelium, where cells showing a morphological similarity to PP M cells (“Villous M cells”) are also acutely (24–48 h) induced at the tips of intestinal villi (45), but without any association with organized mucosal lymphoid tissues or subepithelium dendritic cells. Scanning electron microscopy confirmed the characteristic absence of apical microvilli, while immunohistochemical staining and confocal microscopy showed that these cells only showed one other characteristic of PP M cells: presentation of fucose moieties at the apical surface [bound by the lectin UEA-1, (103)]. These cells significantly deviated from the PP M cell pattern in their expression of M cell specific genes (e.g., gp2, PGRP-S).

There appear to be additional functional subsets of Villous M cells, since other studies show functional transcytosis by sporadically appearing Villous M cells in mice lacking other lymphoid tissues such as PP (104), while cholera toxin-induced Villous M cells appear to lack effective transcytosis function. These other M cells may have yet another distinctive mechanism for induction, with associated distinct functional consequences. Moreover, their distribution is unusual depending on the setting; sporadically appearing Villous M cells are scattered among villous epithelium, while cholera toxin induced Villous M cells appear in broad clusters at the tips of intestinal villi (45).

Villous M cells pose additional puzzles. With cholera toxin, rapid induction at the villous tips clearly rules out their origin from lineage-committed crypt stem cells, and so as with induced NALT M cells, these too fall into the category of transdifferentiation, this time from extant enterocytes. If so, what cellular signals or cytokines are responsible for this rapid conversion? Their apical absence of microvilli still presumably provides an ability to capture luminal microparticles (unpublished data), so what is the purpose of this inducible phenotype? Is it an intestinal epithelium response to microbial invasion (e.g., cholera toxin) to assist in bacterial colonization of the epithelium, or does it provide some as yet unknown benefit to the host? Since these cells are at the tips of the intestinal villi, they are only going to have a brief remaining life span, so is that beneficial to the host? These and other questions need to be addressed, along with a general question to be discussed below on whether inducible M cells change the host-pathogen relationship; that is, do inducible M cells provide any clear benefit to the host or the resident microbes?

## Life History of Inducible M Cells in Chronic Inflammation

In contrast to Villous M cells, we have a slightly clearer picture of the inducing conditions for PP M cell development as discussed above. However, these inducing conditions are not unique to constitutive development of PP nor mucosal organized lymphoid tissues; the cytokines associated with lymphoid tissue induction overlap with cytokines found in chronic inflammation. Chronic production of inflammatory cytokines such as lymphotoxin and TNFα can result in new formation of organized lymphoid tissues. This phenomenon was described as “lymphoid neogenesis” (105), and was often found in models of chronic autoimmune disease. Interestingly, sufficient accumulation of lymphocytes by any means (106, 107) can lead to spontaneous organization of the recruited lymphocyte populations along with induction of stromal cells to help guide the compartmentalization of lymphocytes and dendritic cells [except perhaps, in the brain (108)]. The resulting organized lymphoid tissues are referred to as Tertiary Lymphoid Tissue (TLT); in the intestine, they resemble Isolated Lymphoid Follicles, and might only be distinguished by the conditions leading to their induction.

Chronic inflammation in the intestine, as in Inflammatory Bowel Disease (IBD), microbial infection, and mouse models of these clinical diseases, would therefore seem to provide the basic ingredients for M cell development. Indeed, inflammatory cytokines were found to induce a few M cell-associated genes in intestinal epithelial cell lines such as Caco-2BBe and T84 (86, 87). Caco-2 cell cultures (including co-cultures with B cells) have been a model for M cell differentiation [see for example (49, 109)]; in view of our present knowledge on the diverse phenotypes of M cells, it is not as clear what kind of M cells the induced Caco-2 cells represent, but there are clearly some similarities between induced Caco-2 cells and M cells *in vivo*. This connection has received less attention, as the known effect of inflammatory cytokines on epithelial tight junction function (78, 110, 111) has been a more prominent focus of studies on inflammation and its impact on mucosal barrier function.

Therefore, to study whether chronic inflammation may indeed induce new M cell development, two models of intestinal inflammation were studied. One was an infection by *Citrobacter* that produces a limited infectious colitis, and the other was the Dextran Sodium Sulfate (DSS) model, in which DSS in the drinking water results in epithelial barrier disruption and chronic inflammation (96, 112). In both models, colonic lamina propria inflammation was induced, and M cell numbers were significantly increased. In the DSS model, the M cell induction was dependent on TNFα, as anti-TNF antibodies abrogated this induction. Interestingly, blockade of the LTβR had no effect on M cell development despite significant reduction in RANKL expression, potentially distinguishing this inflammation-associated M cell induction from constitutive LTβR-dependent lymphoid tissue M cell development (96). This effect appears to contrast with the LTβR-independent expression of RANKL/TRANCE by stromal cells in what we would label as “constitutive” mucosal lymphoid tissues (92). On that point, it should be noted that while RANKL was significantly reduced with LTβR blockade in the DSS study, it was not entirely eliminated. Thus, there may be at least some “necessary and sufficient” role of RANKL in both constitutive and inducible M cell development, though synergies with cytokines such as TNF (33, 113) may be variably important.

The DSS-induced M cells were mainly associated with loose mononuclear aggregates, including cells in the subepithelial zone expressing RANKL (96). These aggregates were not fully mature organized lymphoid tissues, as immunostaining for CXCL13 and ER-TR7 showed only minimal induction of these stromal cell markers (96, 112). In another model of intestinal inflammation where a deletion of a TNFα regulatory sequence results in excess TNFα production (TNF^Δ*ARE*^) (114), the ileum showed extensive lamina propria mononuclear inflammation and development of TLT (115, 116). As with the DSS model, we expect strong induction of new M cells accompanying the mononuclear infiltrates, whether associated with TLT or less organized lymphoid aggregates.

Thus, chronic inflammation has been associated with induction of TLT, and so predictably, in mucosal tissues, inflammation is also associated with M cell induction. While organized lymphoid tissues will augment the constitutive organized mucosal lymphoid tissues, there is clearly M cell induction without fully formed or organized lymphoid tissue. The consequences of this induction are implied by the discussion above on the gauntlet provided by subepithelial dendritic cells and B cells. That is, in the loosely aggregated infiltrates, induced M cells may actually contribute to pathogenesis through transcytosis of luminal microbes bypassing any gauntlet, promoting free access of bacteria to the lamina propria, and innate immune signaling and inflammatory cell recruitment. In this context, while induction of TLT may add to the organized lymphoid component, the associated M cell activity in disorganized infiltrates may in fact be paradoxically responsible for driving disease pathogenesis.

## Conclusion: Clever Cell

This discussion has reviewed the remarkable functional features of mucosal M cells that distinguish them from other mucosal epithelial phenotypes; they are cleverly engineered so that they may serve their critical role in immune surveillance of the mucosal lumen. Because the specializations associated with M cell functions discussed here appear to be so novel, there will be much work to do to dissect the molecular details since there are few examples in cell biology to draw from. It is hoped that this discussion will trigger further work on these topics, especially the (incomplete) list provided here:

First, we argue that the M cell is essentially a unique morphological specialization: a strict absence of conventional apical structures (microvilli or cilia). This morphological distinction from canonical enterocytes with apical microvilli and airway epithelium with cilia converges toward a unique morphology, conferring a new functional capability. That is, the shift in the surface electrostatic charge enables the capture of luminal microparticles. This apical specialization is also found in Villous M cells; while they may lack some of the other features of M cells, this apical morphological change may have a purpose involving apical capture of luminal microbes, even in the absence of transcytosis.

Second, M cells have developed a unique cellular machinery to capture large cargo at the apical membrane and transport them to the basolateral end for delivery to dendritic cells. There is no other similar mechanism known among epithelial tissues, and it does not appear to have been borrowed from other phagocytic cell types either. Indeed, if it can be confirmed that M cells have actually re-assigned tight junction proteins to enable large cargo endocytosis, dissection of this machinery may provide additional clues not only to cellular functions, but also to ways in which invasive microbes may have co-evolved with these cellular specializations.

Third, the apparent inducibility of M cell development in intestinal inflammation raises a potential role for M cells in Inflammatory Bowel Disease. While cytokine-mediated damage to tight junction integrity is appealing as a mechanism for leakage of luminal contents into the lamina propria, the specialized active transcytosis by M cells may be a major contributor to pathogenesis. If confirmed, this could suggest new therapeutic strategies to target rogue M cell transcytosis.

This discussion has not covered a few areas where M cells may play a major role, such as the dichotomy of M cells in mucosal tolerance vs. immunity; evidence to date suggests that M cells do not provide any adjuvant activity and are only neutral antigen delivery providers that can just as easily induce immunological tolerance (13, 14). This neutrality may be important in whether M cell surveillance of the intestinal microbiome plays any role in shaping the microbiome. Finally, there is still more to learn about the biology of M cells in immune surveillance in the lung airways. However, this only emphasizes the fact that studies on M cell biology are only beginning to pick up speed, and the role of M cells will be much more than a minor mention in the basic immunology textbooks.

## Author Contributions

All authors listed have made a substantial, direct and intellectual contribution to the work, and approved it for publication.

### Conflict of Interest Statement

The authors declare that the research was conducted in the absence of any commercial or financial relationships that could be construed as a potential conflict of interest.
